# Differences in HIV Natural History among African and Non-African Seroconverters in Europe and Seroconverters in Sub-Saharan Africa

**DOI:** 10.1371/journal.pone.0032369

**Published:** 2012-03-06

**Authors:** Nikos Pantazis, Charles Morrison, Pauli N. Amornkul, Charlotte Lewden, Robert A. Salata, Albert Minga, Tsungai Chipato, Harold Jaffe, Shabir Lakhi, Etienne Karita, Kholoud Porter, Laurence Meyer, Giota Touloumi, on behalf of CASCADE Collaboration in EuroCoord and ANRS 1220 Primo-CI Study Group

**Affiliations:** 1 Department of Hygiene, Epidemiology and Medical Statistics, University of Athens Medical School, Athens, Greece; 2 FHI 360, Durham, North Carolina, United States of America; 3 International AIDS Vaccine Initiative (IAVI), San Francisco, California, United States of America; 4 Université Victor Segalen Bordeaux 2, ISPED, Bordeaux, France; 5 Case Western Reserve University, Cleveland, Ohio, United States of America; 6 Programme PAC-CI, Abidjan, Côte d'Ivoire; 7 University of Zimbabwe, Harare, Zimbabwe; 8 University of Oxford, Oxford, United Kingdom; 9 Zambia Emory HIV Research Project (ZEHRP), Lusaka, Zambia; 10 Project San Francisco, Kigali, Rwanda; 11 Medical Research Council Clinical Trials Unit, London, United Kingdom; 12 Inserm, U1018, HIV Epidemiology, Université Paris-Sud, AP-HP, Le Kremlin-Bicêtre, France; 13 Inserm, U897, Bordeaux, France; Institute of Infectious Diseases and Molecular Medicine, South Africa

## Abstract

**Introduction:**

It is unknown whether HIV treatment guidelines, based on resource-rich country cohorts, are applicable to African populations.

**Methods:**

We estimated CD4 cell loss in ART-naïve, AIDS-free individuals using mixed models allowing for random intercept and slope, and time from seroconversion to clinical AIDS, death and antiretroviral therapy (ART) initiation by survival methods. Using CASCADE data from 20 European and 3 sub-Saharan African (SSA) cohorts of heterosexually-infected individuals, aged ≥15 years, infected ≥2000, we compared estimates between non-African Europeans, Africans in Europe, and Africans in SSA.

**Results:**

Of 1,959 (913 non-Africans, 302 Europeans - African origin, 744 SSA), two-thirds were female; median age at seroconversion was 31 years. Individuals in SSA progressed faster to clinical AIDS but not to death or non-TB AIDS. They also initiated ART later than Europeans and at lower CD4 cell counts. In adjusted models, Africans (especially from Europe) had lower CD4 counts at seroconversion and slower CD4 decline than non-African Europeans. Median (95% CI) CD4 count at seroconversion for a 15–29 year old woman was 607 (588–627) (non-African European), 469 (442–497) (European - African origin) and 570 (551–589) (SSA) cells/µL with respective CD4 decline during the first 4 years of 259 (228–289), 155 (110–200), and 199 (174–224) cells/µL (p<0.01).

**Discussion:**

Despite differences in CD4 cell count evolution, death and non-TB AIDS rates were similar across study groups. It is therefore prudent to apply current ART guidelines from resource-rich countries to African populations.

## Introduction

HIV disease progression is characterized by CD4 cell depletion leading to severe immunodeficiency and death in the absence of effective treatment [1], [2]. CD4 cell count, along with plasma HIV-RNA have been established as the most important prognostic markers of HIV-1 disease progression [3], [4], [5] and, as such, are used to guide therapeutic decisions [6].

Current treatment guidelines are largely based on data from high-income countries, although the vast majority of the world's HIV-infected people live in low and middle-income countries, particularly sub-Saharan Africa (SSA) [7]. A limited number of studies have estimated CD4 cell loss in ART-naïve individuals in African countries [8], [9], [10], and a few have directly compared this to estimates derived from high-income countries [11], [12], [13], [14]. Fewer still have used data from individuals with well-estimated dates of HIV seroconversion [12], [14]. This is important as measures of CD4 cell count from seroprevalent HIV cohorts do not capture duration of HIV infection sufficiently [15]. Furthermore, no study has directly compared time from HIV seroconversion to treatment initiation, clinical AIDS (i.e. not including CD4<200 cells//µL) [16], or death in SSA and high-income countries. It remains crucial to understand whether any observed differences in the rate of CD4 cell decline between population groups leads to appreciable survival differences. Such differences would need to be considered when developing guidelines on the optimal timing of treatment initiation. In addition, understanding population-specific differences in CD4 cell levels and dynamics after seroconversion could guide study designs in evaluating multi-national HIV prevention and vaccine efficacy trial endpoints.

The objectives of this study are to assess and compare CD4 cell trends, from the time of HIV seroconversion, but prior to antiretroviral treatment initiation, and time to clinical AIDS or death, in persons followed in SSA and European cohorts. We also compare differences in CD4 cell decline and time-to-events between Africans living in Europe with those living in Africa. Individuals in the former group were likely infected in Europe as they had a previous negative HIV test documented in a European clinic.

## Methods

### Ethics Statement

All collaborating cohorts received approval from their respective or national ethics review boards. Ethics approval for CASCADE collaborating cohorts has been granted by the following committees: Austrian HIV Cohort Study: Ethik-Kommission der Medizinischen Universität Wien, Medizinische Universität Graz – Ethikkommission, Ethikkommission der Medizinischen Universität Innsbruck, Ethikkommission des Landes Oberösterreich, Ethikkommission für das Bundesland Salzburg; PHAEDRA cohort: St Vincent's Hospital, Human Research Ethics Committee; Southern Alberta Clinic Cohort: Conjoint Health Research Ethics Board of the Faculties of Medicine, Nursing and Kinesiology, University of Calgary; Aquitaine Cohort: Commission Nationale de l'Informatique et des Libertés; French Hospital Database: Commission nationale de l'informatique et des libertés CNIL; French ANRS PRIMO Cohort: Comité Consultatif de Protection des Personnes dans la Recherche Biomédicale; French ANRS SEROCO Cohort: Commission Nationale de l'Informatique et des Libertés (CNIL); German HIV-1 Seroconverter Study: Charité, University Medicine Berlin; AMACS: Bioethics & Deontology Committee of Athens University Medical School and the National Organization of Medicines; Greek Haemophilia Cohort: Bioethics & Deontology Committee of Athens University Medical School and the National Organization of Medicines; ICoNA cohort: San Paolo Hospital Ethic Committee; Italian Seroconversion Study: Comitato etico dell'Istituto Superiore di Sanità; Amsterdam Cohort Studies in Homosexual Men and IDUs: Academic Medical Centre, University of Amsterdam; Oslo and Ulleval Hospital Cohorts: Regional komite for medisinsk forskningsetikk - Øst- Norge (REK 1); Badalona IDU Hospital Cohort: Comité Ético de Investigación Clínica del Hospital Universitari Germans Trias i Pujol; CoRIS-scv: Comité Ético de Investigación Clínica de La Rioja; Madrid Cohort: Ethics Committee of Universidad Miguel Hernandez de Elche; Valencia IDU Cohort: Comité Etico de Investigación Clínica del Hospital Dr. Peset-Valencia; Swiss HIV Cohort Study: Kantonale Ethikkommission, spezialisierte Unterkommission Innere Medizin, Ethikkommission beider Basel, Kantonale Ethikkommission Bern, Comité départemental d'éthique de médecine et médecine communautaire, Commission d'éthique de la recherche clinique, Université de Lausanne, Comitato etico cantonale, Ethikkommission des Kantons St.Gallen; UK Register of HIV Seroconverters: South Birmigham REC; Early Infection Cohorts: Kenya Medical Research Institute, Kenyatta National Hospital, Uganda Virus Research Institute Science and Ethics Committee, Uganda National Council for Science and Technology, Uganda Virus Research Institute Science and Ethics Committee, Uganda National Council for Science and Technology, University of Zambia Research Ethics Committee, Emory IRB, National Ethics Committee of Rwanda, University of Cape Town Research Ethics Committee, University of KwaZulu-Natal Nelson R Mandela School of Medicine; Genital Shedding Study Cohort: University Hospitals of Cleveland, IRB for Human Investigation (CWRU), AIDS Research Committee (ARC), STD/AIDS Control Programme, Uganda Ministry of Health, Committee on Human Research (CHR), Office of Research Administration (UCSF), Biomedical Research & Training Institute (BRTI) – Zimbabwe, Institutional Review Office, Fred Hutchinson Cancer Research Center, Medical Research Council of Zimbabwe (MRCZ). Written informed consent was obtained from all participants. Minors are not included.

### Patients

We used data from the CASCADE Collaboration (Concerted Action of Seroconversion to AIDS and Death in Europe) in EuroCoord (www.EuroCoord.net) and the ANRS 1220 PRIMO-CI study. CASCADE is a collaboration of 25 cohorts of individuals with well-estimated dates of HIV seroconversion (seroconverters) [17]; PRIMO-CI is an ongoing prospective cohort of HIV-1 seroconverters initiated in June 1997 in Abidjan, Côte d'Ivoire [18]. At the time of data update in September 2009 CASCADE included 20 European, 2 Australian and one Canadian cohort. Two SSA cohorts have also joined CASCADE: the International AIDS Vaccine Initiative (IAVI)-sponsored African HIV Research Network Early HIV Infection Cohort (Kenya, Uganda, Rwanda, Zambia, South Africa) [19] and the FHI-sponsored Hormonal Contraception (HC) and HIV Genital Shedding and Disease Progression (GS) Cohort in Uganda and Zimbabwe [20], [21]. Data from the Canadian and Australian CASCADE cohorts were excluded from this analysis.

The seroconversion date is estimated by various methods: most frequently as the midpoint between the last documented negative and first positive HIV antibody test dates with an interval of less than 3 years between tests, through laboratory evidence of seroconversion (PCR positivity in the absence of HIV antibodies or antigen positivity with fewer than four bands on Western blot), or as the date of a seroconversion illness with both an earlier documented negative and a later positive HIV test not more than 3 years apart.

Given that the vast majority of individuals recruited in the SSA cohorts were infected heterosexually and seroconverted in or after the year 2000, individuals infected through other modes of transmission or before year 2000 were excluded from current analyses. Since patterns of change in CD4 count may differ for children, individuals aged less than 15 years at seroconversion were also excluded. Finally, we excluded all CD4 cell measurements taken after combination antiretroviral therapy (cART) initiation or clinical AIDS development and used data only from individuals with at least two eligible CD4 cell measurements. cART was defined as either a protease inhibitor-based or a non-nucleoside reverse transcriptase inhibitor-based regimen, in combination with at least two nucleoside or nucleotide reverse transcriptase inhibitors (NRTIs), or a triple NRTI regimen. Women receiving single dose nevirapine for prevention of mother-to-child HIV transmission were not censored.

Eligible individuals were categorized according to the location of their cohort (*i.e.* Europe or SSA). Using information from two variables collected in CASCADE: “ethnicity” and “geographic origin”, we further divided individuals belonging to European cohorts. Because limited ethnicity data were collected in the European cohorts, individuals who were non-white, and whose country of origin was in SSA or North Africa, were classified as “Europeans - African origin”. All other seroconverters in European cohorts were classified as “non-African Europeans”. Three groups were thus created: non-African Europeans, Europeans - African origin, and SSAs.

### Statistical analysis

CD4 counts at cART initiation and at AIDS diagnosis were estimated through a modified Kaplan–Meier technique [22]. We estimated average CD4 cell trends over time, accounting for the correlation among repeated measurements within each individual, through linear mixed models with random intercept and random slope [23]. We used the square root transformation of CD4 count values to normalise their distribution, stabilise their variance and linearise changes over time [24].

Using Kaplan-Meier methods, we also estimated times from HIV seroconversion to each of the following: clinical AIDS, initiation of cART, death, and loss to follow-up adjusting for late entry into the cohort. In order to assess whether differences in time to clinical AIDS were due to pulmonary or extra-pulmonary tuberculosis (TB), an AIDS-defining event known to occur at higher rates in African compared to European populations, we repeated the analysis excluding TB as an AIDS event. Loss to follow-up was defined as a gap of more than 12 (or 6 in a sensitivity analysis) months between a patient's last CD4 evaluation or clinical assessment and the date their cohort dataset was pooled within CASCADE.

Sensitivity analyses were performed to investigate the robustness of our main findings, taking into account the potentially informative truncation [25], [26] of CD4 cell measurements due to cART initiation or loss to follow-up. More specifically, our final multivariable mixed model was refitted using joint models for both the evolution of CD4 cell counts and the time to the potentially informative event (i.e. cART initiation or loss to follow-up). The proportion of individuals who progressed to AIDS or died while ART-naïve was negligible, thus AIDS and death were not considered as informative censoring events. However, to minimize the potential bias that may result from those censoring mechanisms, the analysis was repeated with follow-up censored at 3 years following seroconversion. Finally, sensitivity analyses included the application of the main multivariable mixed model on a dataset where individuals with only one eligible CD4 cell measurement were included.

All analyses were performed using Stata 10.1 (StataCorp, TX USA). Joint models were fitted through the Joint Multivariate Random Effects (JMRE) [26] method using the user-written *jmre1* command in Stata [27].

## Results

### Study population characteristics

The CASCADE database included data from 20,631 individuals: 19,921 from Europe, Australia or Canada, 408 from IAVI-sponsored cohorts and 302 from FHI-sponsored studies. Data from a further 285 individuals from the Primo-CI cohort were also included.

Of the 19,921 individuals from resource-rich countries, we excluded: 12,910 who had seroconverted before 2000, 4,901 who were not infected through sex between men and women, 519 who had one or no CD4 counts while ART-naïve and AIDS-free, 374 who were from non-European cohorts, and 2 who were aged <15 years. From the 995 individuals in the three SSA cohorts, we excluded 251 individuals: 105 had seroconverted pre-2000, 94 were not infected through sex between men and women, and 52 had one or no CD4 counts while ART-naïve and AIDS-free. In summary, the three study groups in these analyses comprising 1,959 individuals were: non-African Europeans (N = 913), Europeans - African origin (N = 302), and SSAs (N = 744).

Of the 1,959 individuals in the final analysis, two-thirds were female; the median age at seroconversion was 31 years (Table 1). The median (IQR) duration of follow-up was 3.5 years (1.8–5.7), representing a total of 7,420 person-years (Table 2). The median number of CD4 measurements was seven and was higher in SSAs (10) than in the non-African Europeans (5) and Europeans - African origin (4).

**Table 1:** Demographic and clinical characteristics of 1959 seroconverters from European and sub-Saharan African (SSA) cohorts.

	Group				
	Europe (non-African origin) N = 913	Europe (African origin) N = 302	SSA N = 744	Overall N = 1,959	P
Sex					<0.001
*Male*	375 (41.1)	87 (28.8)	237 (31.9)	699 (35.7)	
*Female*	538 (58.9)	215 (71.2)	507 (68.1)	1260 (64.3)	
Median (IQR) age at SC (years)	34 (26, 44)	29 (25, 35)	29 (24, 34)	31 (25, 38)	<0.001
Ethnic group					<0.001
*White*	243 (26.6)	0 (0.0)	0 (0.0)	243 (12.4)	
*Black*	53 (5.8)	39 (12.9)	744 (100.0)	836 (42.7)	
*Other*	9 (1.0)	0 (0.0)	0 (0.0)	9 (0.5)	
*Unknown*	608 (66.6)	263 (87.1)	0 (0.0)	871 (44.5)	
Initiated cART	483 (52.9)	181 (59.9)	167 (22.4)	831 (42.4)	<0.001
Developed AIDS	32 (3.5)	13 (4.3)	58 (7.8)	103 (5.3)	0.002
Died	15 (1.6)	3 (1.0)	23 (3.1)	41 (2.1)	0.177
Year of SC					<0.001
*2000–03*	560 (61.3)	170 (56.3)	314 (42.2)	1044 (53.3)	
*2004+*	353 (38.7)	132 (43.7)	430 (57.8)	915 (46.7)	
Method of SC determination					0.001
*Midpoint*	785 (86.0)	263 (87.1)	683 (91.8)	1731 (88.4)	
*Lab. evidence*	121 (13.3)	36 (11.9)	61 (8.2)	218 (11.1)	
*SC illness*	7 (0.8)	3 (1.0)	0 (0.0)	10 (0.5)	
Subtype					<0.001
*A*	14 (1.5)	1 (0.3)	156 (21.0)	171 (8.7)	
*B*	81 (8.9)	2 (0.7)	2 (0.3)	85 (4.3)	
*C*	26 (2.8)	1 (0.3)	312 (41.9)	339 (17.3)	
*CRF01*	12 (1.3)	0 (0.0)	0 (0.0)	12 (0.6)	
*CRF02*	22 (2.4)	29 (9.6)	67 (9.0)	118 (6.0)	
*Other*	10 (1.1)	9 (3.0)	93 (12.5)	112 (5.7)	
*Unknown*	748 (81.9)	260 (86.1)	114 (15.3)	1122 (57.3)	
Median (IQR) time (months)	5.8	7.2	3.7	5.2	<0.001
between SC and enrolment	(2.1, 10.9)	(3.6, 12.0)	(0.6, 7.8)	(2.0, 10.0)	
Median (IQR) time (months)	10.7	14.0	2.9	6.6	<0.001
between last HIV− and HIV+ test*	(5.2, 19.0)	(8.2, 22.0)	(1.4, 4.2)	(2.9, 15.0)	

Numbers and percentage of respective populations given unless otherwise indicated;

* Only for those with a “Midpoint” method of SC determination; IQR: Interquartile Range; SC: Seroconversion.

**Table 2:** Available CD4 cell measurements, frequency and follow-up time of 1959 seroconverters from European and sub-Saharan African (SSA) cohorts.

	Group				
	Europe (non-African origin)	Europe (African origin)	SSA	Overall	
	Median (IQR)	Median (IQR)	Median (IQR)	Median (IQR)	P-value
Time from seroconversion to first CD4 cell measurement (*months*)	6.1 (2.7, 11.2)	7.4 (3.6, 12.1)	3.7 (0.6, 7.8)	5.3 (2.4, 10.1)	<0.001
Time from seroconversion to last available CD4 cell measurement (*years*)	3.5 (2.1, 5.2)	3.4 (2.0, 5.1)	3.5 (1.6, 6.3)	3.5 (1.8, 5.7)	0.566
Time from seroconversion to last CD4 cell measurement after applying truncation* (*years*)	2.1 (1.1, 3.5)	1.6 (1.0, 3.1)	2.9 (1.4, 5.4)	2.3 (1.2, 4.0)	<0.001
Interval between consecutive CD4 cell measurements (*months*)	3.2 (2.3, 4.6)	2.9 (1.6, 4.4)	2.9 (2.4, 3.9)	3.0 (2.3, 4.4)	0.001
Number of CD4 cell measurements	5 (3, 9)	4 (2, 8)	10 (6, 15)	7 (3, 11)	<0.001

* CD4 cell measurements after cART initiation or AIDS onset were truncated for this analysis; IQR: Interquartile Range.

### CD4 cell count evolution

Both the unadjusted and adjusted models estimate lower initial CD4 cell count as well as slower rates of decline for all Africans compared to their non-African European counterparts (Table 3). Differences between the unadjusted and adjusted model were small with the group effects on the rate of CD4 cell decline being slightly attenuated in the adjusted models.

**Table 3:** Estimates from fitting a univariate (unadjusted) and a multivariable (adjusted) linear mixed model on all repeated measurements of CD4 cell counts (square root transformed) taken from seroconversion to end of follow-up, cART initiation or clinical AIDS onset.

	Unadjusted			Adjusted		
Covariate	Coef.^1^	95% C.I.	p-value	Coef.^1^	95% C.I.	p-value
**CD4 cell count at seroconversion (** ***square root cells/µL*** **)**						
Reference category^2^	24.20	(23.85, 24.55)	<0.001	23.53	(23.08, 23.97)	<0.001
Group						
*Europe (non-African origin)* *	0.00			0.00		
*Europe (African origin)*	−2.83	(−3.55, −2.11)	<0.001	−2.97	(−3.69, −2.25)	<0.001
*SSA*	−0.68	(−1.20, −0.17)	0.009	−0.76	(−1.28, −0.25)	0.004
Sex						
*Male* *	—	—	—	0.00		
*Female*	—	—	—	1.11	(0.65, 1.57)	<0.001
**CD4 cell rate of decline (** ***square root cells/µL per year*** **)**						
Reference category^3^	−1.56	(−1.69, −1.42)	<0.001	−1.49	(−1.69, −1.30)	<0.001
Group						
*Europe (non-African origin)* *	0.00			0.00		
*Europe (African origin)*	0.62	(0.32, 0.92)	<0.001	0.51	(0.21, 0.81)	0.001
*SSA*	0.44	(0.25, 0.62)	<0.001	0.34	(0.15, 0.54)	0.001
Age at seroconversion (*years*)						
*15–29* *	—	—	—	0.00		
*30–39*	—	—	—	−0.10	(−0.29, 0.08)	0.284
*40–49*	—	—	—	−0.34	(−0.62, −0.06)	0.016
*50+*	—	—	—	−0.63	(−0.97, −0.30)	<0.001
Enrolment time (*months*)						
*≤6* *	—	—	—			
*>6*	—	—	—	0.22	(0.05, 0.38)	0.010

* Baseline category;

1 : Coefficients denote either mean estimated value for the reference category or mean estimated differences relative to the baseline category;

2 : Men of non-African origin in European cohorts;

3 : Individuals of non-African origin in European cohorts, aged 15–29 years at seroconversion and with a ≤6 months gap between seroconversion and study entry.

Women had consistently higher CD4 cell counts. Younger individuals and those with a delayed cohort entry of more than 6 months had a slower CD4 cell decline. Calendar year of seroconversion, method of seroconversion determination, and interval between last negative and first positive HIV tests dates were not significantly associated with CD4 cell decline, after adjusting for other factors included in the multivariable model.

**Figure 1:**
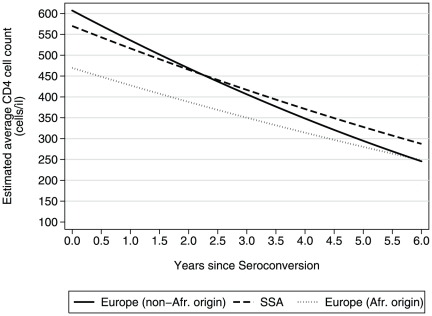
Predicted average CD4 cell count evolution by study group (women, aged 15–29 years at seroconversion, enrolled into cohorts within 6 months of seroconversion).

The mean (95% C.I.) estimated CD4 cell count at seroconversion for women, aged 15–29 years who enrolled into the cohorts within 6 months of seroconversion were 607 (588, 627), 469 (442, 497) and 570 (551, 589) cells/µL for non-African Europeans, Europeans - African origin, and SSA cohorts, respectively (Figure 1). The corresponding CD4 cell loss in the first 4 years from seroconversion was 259 (228, 289), 155 (110, 200) and 199 (174, 224) cells/µL, respectively.

### AIDS diagnoses, survival, cART initiation and loss to follow-up

**Figure 2:**
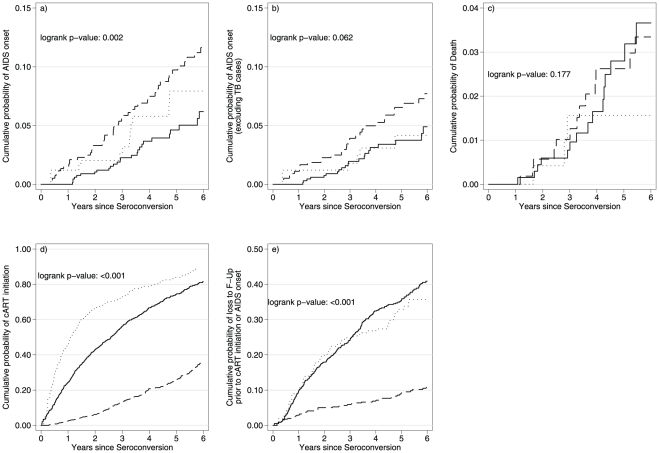
Kaplan-Meier curves of the cumulative probability of a) clinical AIDS, b) clinical AIDS (excluding TB cases) c) Death, d) cART initiation and e) loss to follow-up (≥12 months) before cART initiation or AIDS onset. Curves shown by study group: individuals of non-African origin (solid line) or of African origin (dotted line) in European cohorts and individuals in SSA cohorts (dashed line).

Despite the slower rate of CD4 cell loss, the probability of being diagnosed with clinical AIDS was higher in all Africans, particularly in SSA cohorts (Figure 2). The most common clinical AIDS events in SSA cohorts were pulmonary TB (29.7%) and HIV wasting syndrome (14.9%). The corresponding events in Europe were Pneumocystis pneumonia (20.0%) and Kaposi Sarcoma (17.1%) among persons of non-African origin; TB was the most common first AIDS-defining event (61.6%) among Europeans of Africa origin (extra-pulmonary (38.5%) and pulmonary (23.1%)). After excluding pulmonary or extra-pulmonary TB as an AIDS event, however, we found only a marginal difference in time to clinical AIDS between the three groups (p = 0.06). Overall survival including persons with TB was similar, however, among the three study groups.

Individuals in SSA cohorts tended to initiate treatment later than those in the European cohorts (p<0.001). Estimated median (95% CI) CD4 cell count at cART initiation was 296 (287, 309), 274 (262, 290) and 188 (172, 192) cells/µL for non-African Europeans, Europeans of African origin, and SSAs, respectively (p<0.001). The corresponding estimated median (95% CI) CD4 cell count at AIDS diagnosis was 42 (19, 116), 143 (<52, 164) and 82 (22, 126) cells/µL (p = 0.078).

Rates of loss to follow-up (among AIDS-free persons not on cART) were substantially higher in European cohorts compared to SSA cohorts (Figure 2) (p<0.001).

### Sensitivity analyses

Sensitivity analyses showed that our results were robust when taking into account the potentially informative truncation of CD4 cell measurements due to loss to follow-up. When we simultaneously fitted the final multivariable model for CD4 cell evolution (Table 3) with a survival model for the time to loss to follow-up, the changes in the estimates of the CD4 cell count model were negligible irrespectively of the loss to follow-up definition (i.e. ≥12 or ≥6 months gap).

We adjusted for potentially informative truncation of CD4 cell count measurements due to cART initiation. This resulted in a much faster decline in CD4 count, but the average CD4 cell count at seroconversion and the corresponding differences between groups remained similar to those in the main analysis. The corresponding difference between individuals in SSA and non-African Europeans became more pronounced and was highly significant; the difference in slopes between non-African Europeans and Europeans - African origin became attenuated and was no longer significant. However, treating cART as an informative censoring event is probably inappropriate in this case since decisions about its initiation are mainly based on already observed outcomes (i.e. prior CD4 cell measurements). Moreover, the application of the mixed models in our main analysis on data where we artificially censored follow-up at 3 years after seroconversion, yielded very similar estimates to those reported in Table 3.

Finally, when we included data from the 469 individuals with only one CD4 cell count while ART-naïve and AIDS-free, our main findings remained practically unchanged. The sole exception was that the difference in initial CD4 cell count between individuals in SSA and non-African Europeans was attenuated and no longer statistically significant. The majority of the additional individuals were in the latter group and started cART immediately after their first CD4 cell measurement.

## Discussion

We found that Africans, particularly those in European cohorts, have lower initial CD4 cell counts and slower rates of CD4 cell decline in the absence of cART. This does not, however, appear to have conferred them with any survival advantage. That said, because we had limited duration of follow-up (median, 3.5 years), there may be inherent differences in long-term survival that we were unable to detect.

Although seroconverters in SSA were initially seemingly disadvantaged by having lower CD4 cell counts at seroconversion, their subsequent slower CD4 cell loss resulted in them reaching the same CD4 levels as the other two groups by approximately 2.5 years after seroconversion. Their average CD4 count was higher thereafter falling below 350 cells/µL almost 6 months later than that of non-African Europeans. In contrast, Europeans - African origin consistently had the lowest average CD4 counts throughout the follow-up period and reached a CD4 count of350 cells/µL approximately 1 to 1.5 years earlier than non-African Europeans and Africans living in SSA, respectively. Of note, the slower CD4 cell count decline in African populations remained evident even after conditioning on CD4 levels at seroconversion.

These findings are in agreement with two smaller seroconverter cohorts with shorter follow-up time [12], [14] and a number of seroprevalent cohorts, of lower CD4 cell counts and similar or milder CD4 cell decline in Africans compared to their counterparts in Europe and North America [11], [13], [28], [29], [30].

It is well known that, even in healthy individuals, lymphocyte subset values are influenced by various factors including genetics and the environment [31]. Genetic factors such as HLA type can segregate along African tribal lines. In sub-Saharan Africa environmental factors such as parasitic (e.g. malaria, schistosomiasis), respiratory, and gastrointestinal co-morbidities are much more common than in Europe and will likely affect the immune status of residents. Furthermore, it has been proposed that the infecting HIV-1 subtype may also influence CD4 levels [13]. Given that subtype information was unavailable for more than 80% of individuals from Europeans cohorts and, for those with known subtype, the most prevalent subtype among non-African Europeans was B (49%) and, among Africans, non-B (69% CRF_02 and 74% A or C for Europeans- African origin and SSA, respectively) with only 2 SSA seroconverters being infected with subtype B, we were not able to assess the contribution of subtype, in addition to that of ethnicity and geographic origin. A large CD4 difference between Africans and non-Africans followed in the French ANRS PRIMO cohort was found after controlling for subtype [32]. Muller et al's study [29] concluded that the differences they observed in rate of CD4 cell loss among Africans and Europeans in the Swiss HIV cohort were unlikely to be due to environmental factors, since they were all located in Switzerland, or to the infecting HIV-1 subtype, since differences persisted even after controlling for subtype. Their conclusion that these differences were likely due to host genetic factors is further supported by our findings of a slower rate of CD4 cell loss experienced by Africans, in both the European and SSA cohorts compared to non-African Europeans. Environmental factors may still play a role, however, since adaptation to a new environment after relocation takes place gradually over time. Unfortunately, we do not have data on dates of migration to Europe for the Europeans of African origin to allow further exploration. Of note, however, we found that CD4 levels for Europeans- African origin were much lower than those in the SSA cohorts. One reason for this is that African populations are genetically very diverse, and large differences in CD4 levels (both pre- and post-seroconversion) have been observed even among populations living in geographically-close countries [33]. In our study, the majority of the Europeans of African origin live in France and are more likely to originate from North and West Africa rather than from Eastern or Southern Africa, where the majority of the participants in the SSA cohorts originate.

Africans, particularly those in SSA cohorts, progressed to clinical AIDS significantly faster than non-African Europeans (p = 0.002). After excluding both pulmonary and disseminated TB as AIDS-defining events, however, we found little evidence of a difference in time to AIDS between groups (p = 0.06). We acknowledge that the power of this comparison is reduced due to the low number of non-TB AIDS events. However, this suggests that the difference between the Europeans and Africans in European cohorts is largely due to differences in TB rates. This TB rate differential partially, accounts for the differences in progression to AIDS between the SSA and both European groups as well. Any remaining difference in time to AIDS may be due to differences in co-infection rates and existing co-morbidities. For example, malaria is reported to facilitate HIV replication through increased cytokine production and immune cell activation [34] leading to CD4 cell loss and the consequent diagnosis of AIDS-defining conditions. Residual differences in progression to AIDS may also be an artefact of the higher number of CD4 cell measurements taken from SSA cohort participants compared to participants in the resource-rich country cohorts (10 vs. 5 and 4 measurements, respectively). The more frequent CD4 testing would allow an AIDS diagnosis to be made earlier. The fact that TB is often diagnosed at higher CD4 cell counts [35] may also explain why the SSA group (which had higher TB rates) progressed to AIDS more rapidly despite their slower CD4 cell decline.

Not surprisingly, given the differences in HIV treatment guidelines based on CD4 counts and the tendency in Europe to treat primary HIV infection, we found that individuals in European cohorts initiated cART earlier than their SSA counterparts. Individuals in SSA cohorts initiated cART with 86–108 CD4 cells/µL, on average, lower than their counterparts in European cohorts. Despite lower CD4 cell count at cART initiation, after TB is excluded as an AIDS-defining event, estimated time to clinical AIDS for individuals from SSA cohorts is similar to that of Europeans. This suggests that the slower CD4 cell loss may have conferred a clinical benefit counterbalancing lower CD4 counts at seroconversion. It also supports the WHO recommendations for active TB case finding and isoniazid preventive therapy for HIV-infected individuals in Africa [36] to improve clinical prognosis.

Europeans - African origin in this analysis were more likely to initiate cART soon after seroconversion compared to non-African Europeans. This is, most probably, because the majority of identified Africans (n = 288, 95%) in European cohorts were enrolled in France, a country with particular interest in treating primary HIV infection [37]; French cohorts of African and non-African ancestry comprised 48% of the data in our analyses.

Interestingly, individuals enrolled into cohorts within 6 months of seroconversion had significantly faster rates of CD4 cell decline compared to those enrolled later. This suggests that, in cohorts with substantial delayed enrolment, there may exist a preferential inclusion of slow progressors because those progressing more rapidly are less likely to be enrolled into cohorts. It may also be because the rate of CD4 loss is higher around the time of seroconversion, and this rapid drop is more likely to be identified in individuals with shorter enrolment times and more frequent CD4 cell measurements. Of note, the time from infection to cohort enrolment was longer in the European, compared to the SSA cohorts, particularly for individuals of African origin. Even after adjusting for this, however, we found significantly slower CD4 cell loss among Europeans - African origin. Moreover, restricting the analysis to those with short (i.e. <6 or <3 months) enrolment delay did not alter significantly our main findings.

These results were based on the analysis of longitudinal data derived from a large collaboration of prospective cohorts in 7 West European and 7 SSA countries. The large size of the CASCADE database allowed us to restrict the analysis to recent seroconverters and to those heterosexually infected. Importantly, the robustness of our findings regarding the effects of the most common truncation mechanisms and the exclusion of individuals with only one CD4 measurement while ART-naïve and AIDS-free has been investigated through a series of sensitivity analyses. Results from these analyses showed that our findings regarding the lower initial CD4 cell count levels of African immigrants in Europe and the slower rate of CD4 cell loss among SSA seroconverters remained consistent with those of the main analysis.

Our study is based on data from observational cohorts. Despite restricting the European data to include only individuals with the characteristics of African cohorts and adjusting for most of the known prognostic and confounding factors, residual confounding and potential selection bias in cohort enrolment cannot be ruled out. Because the healthcare infrastructure in SSA is limited and quality healthcare difficult to access, the frequent study visits, and resulting increased contact with clinicians, experienced by the SSA cohorts could also influence the health status and outcomes for the SSA cohorts. These factors most likely contributed to the low loss to follow-up rate seen in the SSA cohorts as well. The SSA cohorts provided transportation and/or reimbursement for transportation and time, health care services for acute illnesses, regular counselling, active follow-up of individuals who missed study visits and required frequent study visits. All of these are factors effective in promoting low loss to follow-up rates in resource-poor settings. Furthermore, seroconverters may not be representative of the general HIV-infected population, although we recently showed that estimates of HIV progression derived from seroconverter cohorts are similar to that seen in the general HIV-infected population [38].

In conclusion, although Africans in SSA and Europe demonstrated a slower rate of CD4 cell decline and higher rates of tuberculosis than non-African Europeans, survival rates and AIDS rates, excluding TB, were similar. Given that treatment decisions, particularly in resource-rich countries, are largely based on CD4 levels, understanding initial CD4 counts at seroconversion, subsequent CD4 decline, and the time from seroconversion to key CD4 thresholds (e.g. 350, 200 cells/µL) will impact the timing of ART initiation. Based on these findings, it appears, therefore, that HIV treatment guidelines developed from cohorts in Europe are applicable and appropriate for Africans. Universal access and implementation of such guidelines remains the primary challenge.
